# Effect of prior treatments on post-CDK 4/6 inhibitor survival in hormone receptor-positive breast cancer

**DOI:** 10.1007/s10549-022-06823-w

**Published:** 2022-12-21

**Authors:** Jeffrey Franks, Nicole E. Caston, Ahmed Elkhanany, Travis Gerke, Andres Azuero, Gabrielle B. Rocque

**Affiliations:** 1 Department of Medicine, Division of Hematology and Oncology, University of Alabama at Birmingham, 1808 7th Avenue South 35233 - Boshell Diabetes Building, Birmingham, AL USA; 2 O’Neal Comprehensive Cancer Center, Birmingham, AL USA; 3 The Prostate Cancer Clinical Trials Consortium, New York, NY USA; 4 School of Nursing, University of Alabama at Birmingham, Birmingham, AL USA

**Keywords:** Cyclin-dependent kinase 4/6 inhibitors, Visualization, Duration

## Abstract

**Purpose:**

Multiple treatment options exist for patients with metastatic breast cancer (MBC). However, limited information is available on the impact of prior treatment duration and class on survival outcome for novel therapies, such as cyclin-dependent kinase 4/6 inhibitors (CDK4/6i) for patients with hormone receptor-positive, human epidermal growth factor receptor 2-negative (HR+ HER2−) MBC.

**Methods:**

This study used a nationwide, de-identified electronic health record-derived database to identify women with HR+ HER2− MBC who received at least one CDK 4/6i between 2011 and 2020. Hazard ratios (HR) and 95% confidence intervals (CI) were estimated for the association between prior duration and class of cancer treatment (both early-stage and metastatic) and prior CDK 4/6i survival as well as for those with multiple CDK 4/6i.

**Results:**

Of 5363 patients, the median survival from first CDK 4/6 inhibitor administration was 3.3 years. When compared to patients with no prior treatments, patients with < 1 year of prior treatment duration had a 30% increased hazard of death (HR, 1.30; 95% CI 1.15–1.46), those with 1 to < 3 years a 68% increased hazard of death (HR 1.68; 95% CI 1.49–1.88), and those with 3 or more years a 55% increased hazard of death (HR 1.55; 95% CI 1.36, 1.76). Patients who received prior therapy (endocrine or chemotherapy) before their CDK 4/6i had worse outcomes than those who received no prior therapy. Similar results were seen when comparing patients in the metastatic setting alone. Finally, patients who received a different CDK 4/6i after their first saw a lower hazard of death compared to patients who received subsequent endocrine or chemotherapy after their first CDK 4/6i.

**Conclusion:**

Prior treatment duration and class are associated with a decreased overall survival after CDK 4/6 inhibitor administration. This highlights the importance for clinicians to consider prior treatment and duration in treatment decision-making and for trialists to stratify by these factors when randomizing patients or reporting results of future studies.

**Supplementary Information:**

The online version contains supplementary material available at 10.1007/s10549-022-06823-w.

## Introduction

For patients with metastatic breast cancer (MBC), there are a lack of consensus on the optimal treatment sequencing. Within the NCCN guidelines, over 40 regimens are available across subtypes. In hormone receptor-positive, human epidermal growth factor 2-negative (HR+ HER2−) MBC, the most common subtype accounting for 50–60% of cases, more than 20 treatment regimens are listed [1, 2]. This heterogeneity results in a large number of available options for treatment sequencing. Our previous study found that among 6639 sequences of MBC therapy, there were 3718 rare sequences and only 44% of patients received a sequence that 11 other patients had received [3]. Thus, patients presenting with similar characteristics can receive different guideline-based treatment sequences, which may lead to differing outcomes.

The PALOMA, MONARCH, and MONALEESA trials established cyclin-dependent kinase (CDK) 4/6 inhibitors, Palbociclib, Abemaciclib, and Ribociclib, respectively, as standard of care for patients with HR+ HER2− MBC based on observed improved progression-free survival and overall survival benefits ranging from 9.5 to 20.5 months. [4–6] However, these trials reported minimal information regarding the class or duration of treatments received prior to the treatment of interest. The PALOMA-3 trial evaluated Fulvestrant and Palbociclib reporting the frequency of previous treatments as 1, 2, or ≥ 3 with no discussion regarding previous treatment duration [4]. The MONARCH-3 trial of Abemaciclib only reported yes or no to previous chemotherapy or endocrine therapy [7]. Prior studies have shown that early-line treatments influence the response to later-line treatments [8–11]. Therefore, a better understanding of the impact of prior time on treatment, prior treatment class, and prior use of CDK 4/6 inhibitors is needed to apply these treatments to patients. This study leverages real-world data to evaluate the association between prior treatments (both early stage and metastatic) duration and class (e.g., endocrine therapy, chemotherapy, CDK 4/6 inhibitor) and overall survival using both traditional modeling and a visualization technique. [3]

## Methods

### Study design and sample

This retrospective cohort study used the nationwide, electronic health record (EHR)-derived Flatiron Health de-identified database to include women diagnosed with metastatic breast cancer between 2011 and 2020. Flatiron Health is a longitudinal database composed of de-identified patient-level structured and unstructured data curated from approximately 280 US oncology care sites (~ 800 sites of care), including community cancer practices and academic medical centers [12]. The study population included women with HR+ HER2− MBC from Flatiron Health’s MBC database. Inclusion criteria included biomarker information to identify the HR and HER2 statuses prior to their CDK 4/6 inhibitor initiation and patients must have received a CDK 4/6 inhibitor (Palbociclib, Abemaciclib, or Ribociclib) during their treatment course with either no previous treatment, previous endocrine therapy, or previous chemotherapy. The cohort excluded patients who were male, aged less than 18 years old, had missing cancer subtype or treatment, or had suspected incorrect treatment (e.g., receiving docetaxel and paclitaxel at the same time). This study was approved by the University of Alabama at Birmingham Internal Review Board prior to study conduct and included a waiver of informed consent.

### Variables

#### Outcome and overall survival

The primary outcome for this study is overall survival (OS), defined as time from initiation of the first CDK 4/6 inhibitor to death as a result of any cause. Death was recorded in the Flatiron Health database by aggregating structured and unstructured EHR records, Social Security Death Index, and obituaries. [13]

#### Patient characteristics

Patients’ age was determined by the date of their primary breast cancer diagnosis. Age was then categorized as less than 45, 45 to 54, 55 to 64, 65 to 74, and 75 or older. Similarly, patients’ race or ethnicity was categorized as White, Black, Other, or not documented. Other race included Hispanic or Latino, Asian, American Indian, Alaskan Native, and Pacific Islanders; these races were combined due to small sample sizes in the dataset.

#### Clinical characteristics

Patients' sites of metastasis were categorized as visceral, bone, or lymph node only. Patients with at least one visceral site were considered visceral, those without visceral sites but with at least one bone site were considered bone, and those with only lymph node sites were considered lymph node only. Cancer subtype was determined through hormone receptor (HR, estrogen receptor and progesterone receptor) and human epidermal growth factor receptor 2 (HER2) biomarker tests. Patients were considered positive for a biomarker if any tests were positive.

#### Treatment characterization

Prior treatments were defined as any cancer treatment (early or advanced stage) received prior to a CDK 4/6 inhibitor. Treatments were identified using generic drug names recorded as either administered or ordered alongside treatment start and end dates. Cancer treatment duration was defined as the total duration of all cancer treatments—including targeted, endocrine therapy, and/or chemotherapy—received after the patients’ initial cancer diagnosis and prior to the initiation of the first recorded CDK 4/6 inhibitor. Similarly, prior treatment duration in the metastatic setting was defined as the total duration of cancer treatments from the patients’ metastatic diagnosis date to the first recorded CDK 4/6 inhibitor. Prior treatment durations were categorized as 0 years (i.e., no prior treatment), < 1 year, 1 to < 3 years, and 3 or more years. The type of cancer treatments prior to CDK 4/6 inhibitor initiation were categorized as chemotherapy (taken with or without endocrine and targeted therapies), endocrine therapy (with or without targeted therapies; no chemotherapy), or no prior treatment (frontline CDK 4/6 inhibitor in the metastatic setting). To understand if multiple CDK 4/6 inhibitors are associated with improved survival, a subset of patients with at least one treatment after the CDK4/6 inhibitors was identified. In this subset, patients were categorized as receiving either a second CDK 4/6 inhibitor or another treatment (e.g., chemotherapy (taken with or without therapy) or endocrine therapy without chemotherapy (targeted therapies allowed)).

### Statistical analysis

Descriptive analyses included medians and interquartile ranges (IQRs) for continuous variables or frequencies and percentages for categorical variables. The median OS from the initiation of the first CDK 4/6 inhibitor was calculated using the Kaplan–Meier estimator. The associations between OS and total treatment duration and class prior to CDK 4/6 inhibitor initiation were estimated using hazard ratios (HRs) and 95% confidence intervals (CI) from Cox proportional hazard models. Additional analysis included only prior treatment duration and class in the metastatic setting (i.e., treatments after metastatic diagnosis date and prior to the first CDK 4/6 inhibitor). The models were adjusted for age at diagnosis, race and ethnicity, site of metastasis, and metastatic diagnosis year.

For the analysis comparing OS for patients who received a second CDK 4/6 inhibitor vs. another treatment, two HR estimates were computed: (1) an estimate from a Cox proportional hazard model with a time-dependent indicator variable for second CDK 4/6 and (2) an estimate from a prescription time-distribution matched analysis. The model for the first HR estimate was adjusted for age at diagnosis, race and ethnicity, site of metastasis, prior treatment duration (years), first CDK 4/6 inhibitor type, first CDK 4/6 inhibitor duration, and year of first CDK 4/6 inhibitor. For the matched analysis, propensity scores were computed using a non-linear, non-parametric random forest ensemble modeling those with a second CDK 4/6 inhibitor vs. those with another treatment (not CDK4/6 inhibitor) with the aforementioned control variables. Next, radius matching on the propensity score (radius = 0.001) was conducted in addition to selecting each match by being alive at the time of the respective 2nd CDK 4/6 and having a number of therapy lines after first CDK 4/6 equal or greater to the number of therapy lines from the respective first to second CDK 4/6. Then an HR estimate from the matched data was computed. HRs from 1-to-1, 2-to-1, and 3-to-1 matching runs were computed. Analyses were performed using R, version 4.0.5 and SAS© software, version 9.4 (SAS Institute, Cary, NC).

### Visualization

A graphic displaying a random sample of 150 patients’ treatments and their duration was created and displayed using a novel visualization approach that was developed by our team as seen in a prior publication [3]. The software has been updated and made to be more customizable. To summarize, in this visualization paradigm, patients are represented on the y-axis with treatment time on the x-axis. A color-coded treatment bar represents each treatment class within the patient’s course. CDK 4/6 inhibitors are shown as dark purple, endocrine therapies are shown in teal, chemotherapy in green, and other targeted therapies in orange. Treatment gaps are represented as white space. A Kaplan–Meier curve was overlaid as a function of time from CDK 4/6 initiation (referred to here as time zero) to death or censoring. To assess for using CDK4/6 inhibitors beyond progression, a visualization image was created displaying the second CDK 4/6 inhibitor displayed in light purple. Visualization graphics were created using R, version 4.0.5.

## Results

### Sample demographics

A total of 5391 women diagnosed with HR+ HER2− MBC were eligible for inclusion. The demographic and clinical characteristics of the study participants are shown in Table 1. Patients were most commonly aged 55–64 (29%), White (69%), and had visceral metastasis (70%). In the adjuvant and metastatic setting, 33% had no prior treatment, 37% received endocrine therapy alone, and 30% chemotherapy alone or chemotherapy with endocrine. Of those who received CDK 4/6 inhibitors as first line, Palbociclib was the most commonly prescribed (84%). In the metastatic setting, no prior treatment (49%) was most common prior to a CDK 4/6 inhibitor, followed by hormone therapy alone (34%) and chemotherapy alone or chemotherapy in combination with hormone therapy (17%). Of the 635 (12%) patients receiving multiple CDK4/6 inhibitors, the most common order was Palbociclib followed by Abemaciclib (54%).

**Table 1:** Demographic and clinical characteristics of patients overall and by prior treatment class

Characteristics	Overall (*N* = 5391)	^a^No prior treatment (*n* = 1800)	^a^Prior Endocrine therapy (*n* = 1985)	^a^Prior Chemotherapy (*n* = 1606)
Age				
Less than 45	736 (14)	208 (12)	254 (13)	274 (17)
45–54	1234 (23)	377 (21)	424 (21)	433 (27)
55–64	1559 (29)	554 (31)	509 (26)	496 (31)
65–74	1255 (23)	443 (25)	492 (25)	320 (20)
75 or older	607 (11)	218 (12)	306 (15)	83 (5)
Race				
White	3741 (69)	1189 (66)	1462 (74)	1090 (68)
Black	483 (9)	146 (8)	160 (8)	177 (11)
Other	697 (13)	241 (13)	224 (11)	232 (14)
Not documented	470 (9)	224 (12)	139 (7)	107 (7)
Site of metastasis				
Visceral	3746 (70)	1157 (65)	1339 (68)	1250 (78)
Bone	1551 (29)	612 (34)	615 (31)	324 (20)
Lymph node only	80 (1)	25 (1)	25 (1)	30 (2)
CDK 4/6 inhibitor type				
Palbociclib	4099 (76)	1390 (77)	1489 (75)	1220 (76)
Abemaciclib	367 (7)	116 (6)	118 (6)	133 (8)
Ribociclib	330 (6)	118 (7)	117 (6)	95 (6)
Multiple CDK 4/6	595 (11)	176 (10)	261 (13)	158 (10)
^**a**^Prior treatment duration (years)				
0	1938 (36)	1800 (100)	0(0)	0 (0)
< 1 year	1210 (22)	0(0)	840 (42)	508 (31)
1 to < 3 years	1189 (22)	0(0)	633 (32)	556 (35)
3 or more	1054 (20)	0(0)	512 (26)	542 (34)
First CDK 4/6 inhibitor				
Palbociclib	4538 (84)	1514 (84)	1683 (85)	1341 (84)
Abemaciclib	450 (8)	142 (8)	153 (8)	155 (10)
Ribociclib	403 (8)	144 (8)	149 (8)	110 (7)

All patients were diagnosed with metastatic breast cancer in 2011–2020 and had at least one CDK 4/6 inhibitor within their cancer treatment regimen. Prior treatments included those for early stage and metastatic

“Other” race includes Hispanic or Latino, Asian, American Indian, Alaskan Native, and Pacific Islanders

*CDK* cyclin-dependent kinase;

^a^Prior treatment class and duration indicates any cancer treatment prior to initiation of a CDK 4/6 inhibitor

### Overall survival

The median OS was 4.3 years for patients with no therapy prior to a CDK 4/6 inhibitor, but 3.3 years when including patients with any prior treatments. After adjusting for age at diagnosis, race and ethnicity, site of metastasis, and metastatic diagnosis year for patients in the adjuvant and metastatic setting, compared to patients who have not received any treatment, patients with a prior treatment duration of < 1 year had a 30% increased hazard of death (HR, 1.30; 95% CI 1.15, 1.46), those with a prior treatment duration of 1 to < 3 years had a 68% increased hazard of death (HR 1.68; 95% CI 1.49, 1.88), and those with a prior treatment duration of 3 or more years had a 54% increased hazard of death (HR 1.54; 95% CI 1.36, 1.76; Table 2). For patients in the metastatic setting only, compared to patients who received no prior treatment, those who received a prior treatment duration of < 1 year had a 25% increased hazard of death (HR, 1.25; 95% CI 1.13, 1.39), those with a prior treatment duration of 1 to < 3 years had a 39% increased hazard of death (HR 1.39; 95% CI 1.21, 1.61), and those with a prior treatment duration of 3 or more years had a 9% increased hazard of death (HR 1.09; 95% CI 0.87, 1.36). Similar results were seen when analyzing the class of treatment received prior to CDK 4/6 inhibitor administration. In the adjuvant and metastatic setting, patients receiving prior endocrine therapy had a 29% increased hazard of death (HR, 1.29; 95% CI 1.16, 1.44), while patients receiving prior chemotherapy had a 72% increased hazard of death (HR, 1.72; 95% CI 1.54, 1.93) when compared with patients who did not receive a prior treatment. In the metastatic setting, patients receiving prior endocrine therapy had a 23% increased hazard of death (HR, 1.23; 95% CI 1.11, 1.36), while patients receiving prior chemotherapy had a 57% increased hazard of death (HR, 1.57; 95% CI 1.39, 1.78) when compared with patients who did not receive a prior treatment.

**Table 2:** The association between OS and chemotherapy duration and treatment class before CDK 4/6 inhibitor initiation

Prior treatment characteristics	^a^Adjuvant and metastatic setting		Metastatic setting only	
	Adjusted hazard ratio	95% Confidence interval	Adjusted hazard ratio	95% Confidence interval
Treatment duration (years)				
0	–	–	–	–
< 1 year	1.30	1.15 to 1.46	1.25	1.13 to 1.39
1 to < 3 years	1.68	1.49 to 1.88	1.39	1.21 to 1.61
3 or more	1.55	1.36 to 1.76	1.09	0.87 to 1.36
Prior cancer treatment class				
No prior cancer treatment	–	–	–	–
Chemotherapy	1.72	1.54 to 1.93	1.57	1.39 to 1.78
Endocrine therapy	1.29	1.16 to 1.44	1.23	1.11 to 1.36

*N *= 5391

All patients were diagnosed with metastatic breast cancer in 2011–2020. Hazard ratios and 95% confidence intervals obtained by Cox proportional hazard models. Model adjusted for age at diagnosis, race, site of metastasis, and metastatic diagnosis year

*OS* Overall survival; *CDK* cyclin-dependent kinase

^a^Prior treatments included those for early stage and metastatic

The data contained *n* = 595 patients who received a second CDK 4/6 inhibitor and *n* = 2926 who received treatments other than a second CDK 4/6 inhibitors in the subsequent line. Table 3 shows HRs comparing OS for patients who received a second CDK 4/6 inhibitor vs. endocrine, chemotherapy, or other non-CDK4/6-targeted therapies. For the time-dependent analysis, patients who received another CDK 4/6 inhibitor after their first CDK 4/6 inhibitor had a 17% decreased hazard of death when compared with patients who received a single CDK 4/6 inhibitor (HR, 0.83; 95% CI 0.71, 0.96). Additional matched analysis revealed that patients who received another CDK 4/6 inhibitor after their first CDK 4/6 inhibitor had a 21% decreased hazard of death when compared with patients who received only a single CDK 4/6 inhibitor (HR, 0.79; 95% CI 0.65, 0.95). Similar results were seen when 1-to-1 and 1-to-2 matching was analyzed (Supplemental Table 1).

**Table 3:** The association between overall survival and treatment type following the first CDK 4/6 inhibitor

Treatment following first CDK	Hazard ratio	95% Confidence interval
Time-dependent analysis (*n = 3521)		
Single CDK 4/6 inhibitor	–	–
Multiple CDK 4/6 inhibitor	0.83	0.71 to 0.96
Matched analysis (**n = 1500)		
Single CDK 4/6 inhibitor	–	–
Multiple CDK 4/6 inhibitor	0.79	0.65 to 0.95

All patients were diagnosed with metastatic breast cancer in 2011–2020. Hazard ratios and 95% confidence intervals obtained by Cox proportional hazard models. Time-dependent analysis included adjustments for age at diagnosis, race, site of metastasis, first CDK 4/6 inhibitor type, first CDK 4/6 inhibitor duration, and year of first CDK 4/6 inhibitor. Propensity scores were created from age at diagnosis; race; site of metastasis; prior treatment duration (years); first CDK 4/6 inhibitor type; and first CDK 4/6 inhibitor duration

*CDK* cyclin-dependent kinase

^*^Patients were only included if they switched treatments after their first CDK 4/6 inhibitor

^**^ Patients were three-to-one radius matched with *n *= 1125 patients who switched to a later-line endocrine or chemotherapy and *n* = 375 patients with a second CDK 4/6 inhibitor

### Treatment sequence visualization

**Fig. 1:**
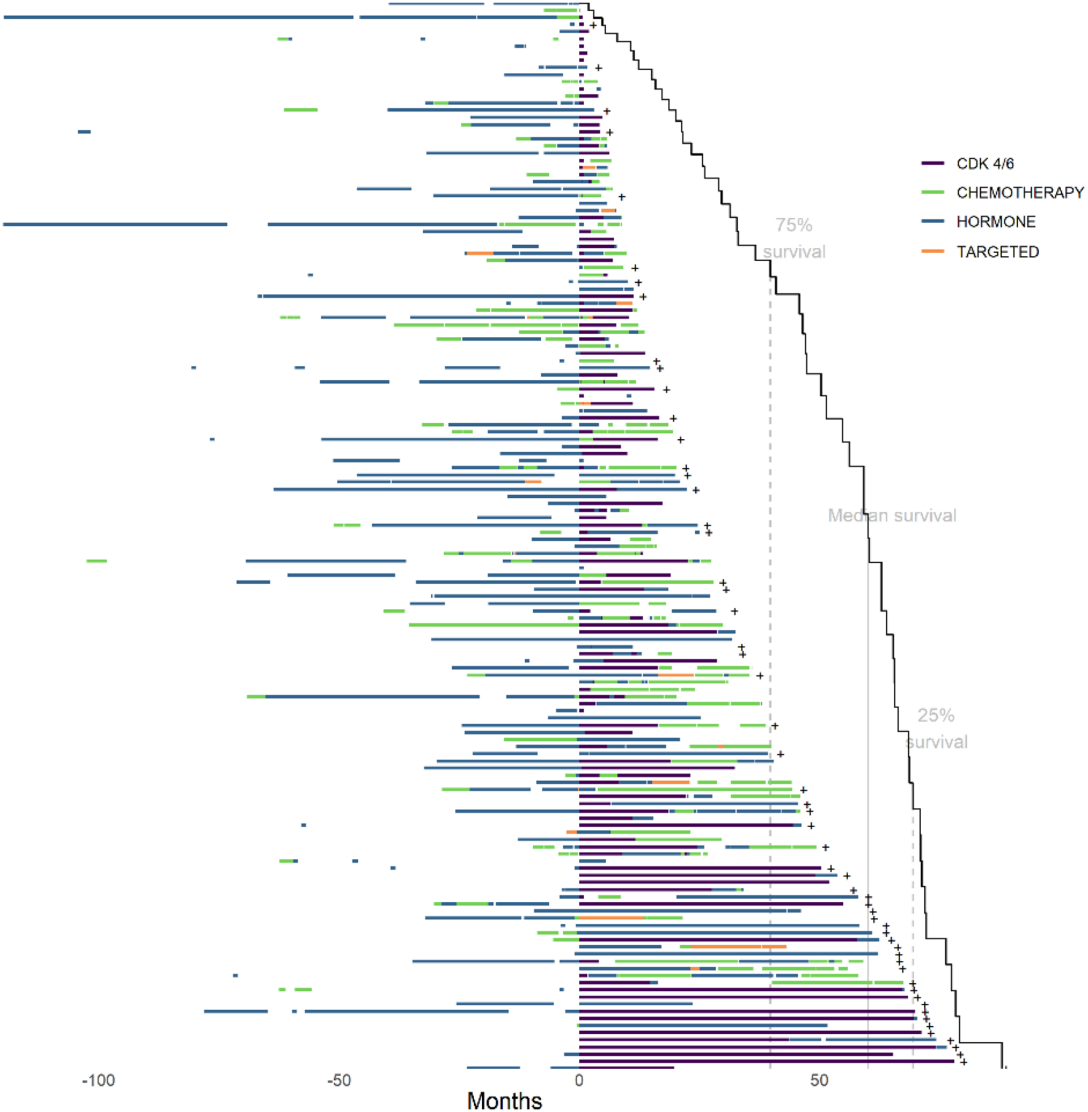
Treatment course of patients diagnosed with metastatic breast cancer who received a CDK 4/6 inhibitor. Definitions: CDK 4/6 inhibitor, cyclin-dependent kinase 4/6; HER2, human epidermal growth factor receptor 2. Random sample of 150 patients from our cohort in 2015. The x-axis includes time in years with the patients first CDK 4/6 inhibitor at time zero. The y-axis includes individual patients. The image is sorted according to overall survival from CDK 4/6 inhibitor initiation. Patients higher on the x-axis have lower survival post-CDK 4/6 inhibitor initiation than those at the bottom. Patients who received endocrine (teal) or chemotherapy (shown in green) prior to CDK 4/6 inhibitor initiation had lower survival compared with those who received CDK 4/6 inhibitors earlier in their treatment course. (Color figure online)

**Fig. 2:**
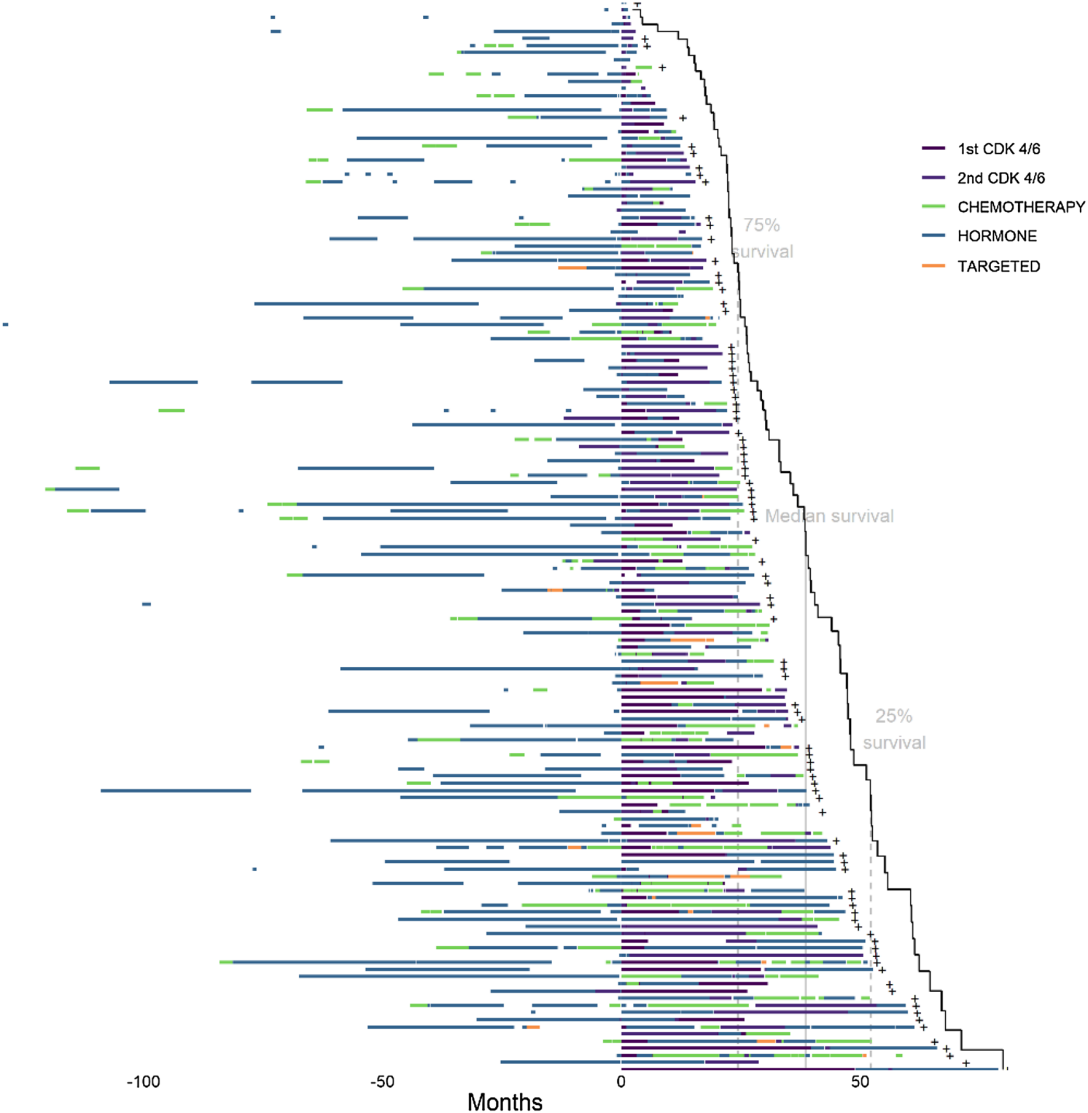
Treatment course of patients diagnosed with metastatic breast cancer who received multiple CDK 4/6 inhibitors. Definition: CDK 4/6 inhibitor, cyclin-dependent kinase 4/6; HER2, human epidermal growth factor receptor 2. Random sample of 150 patients from our cohort. The x-axis includes time in years with the patients first CDK 4/6 inhibitor at time zero. The y-axis includes individual patients. The image is sorted according to overall survival from the first CDK 4/6 inhibitor. Patients higher on the x-axis have lower survival post-CDK 4/6 inhibitor initiation than those at the bottom. Patients with longer first-line CDK 4/6 inhibitors appear to have longer survival and a better response when compared to patients with later-line CDK 4/6 inhibitors

Patients who received endocrine (shown in teal) or chemotherapy (shown in green) prior to CDK 4/6 inhibitor initiation had lower survival compared with those who received CDK 4/6 inhibitors earlier in their treatment course (Fig. 1). Patients near the top of the image have longer treatments prior to first CDK 4/6 inhibitor but show shorter post-CDK 4/6 inhibitor survival compared with those at the bottom of the figure with fewer treatments prior to first CDK 4/6 inhibitor. The figure also displays that patients near the bottom of the image had a similar total duration of any treatment to the patients near the top of the image**. **Figure 2 displays patients who received multiple CDK 4/6 inhibitors throughout their treatment course with the first CDK 4/6 inhibitor being time 0. The initial CDK 4/6 inhibitor is shown in dark purple with the subsequent CDK 4/6 inhibitors shown in lighter purple. Patients with longer first-line CDK 4/6 inhibitors appear to have longer survival and a better response when compared to patients with later-line CDK 4/6 inhibitors.

## Discussion

This study demonstrates the importance of prior treatment duration and class to the initiation of a CDK 4/6 inhibitors in women with HR+ HER2– MBC. Patients with longer cancer treatment durations prior to their first CDK 4/6 inhibitor had an increased hazard of death after their CDK 4/6 inhibitor initiation. This finding was particularly noteworthy in patients that received prior chemotherapy. A study evaluating patients receiving Palbociclib and Letrozole demonstrated that patients without prior cancer treatments who received this regimen had a two-fold PFS benefit compared to those with prior endocrine therapy or chemotherapy [14]. Similarly, an analysis of patients with MBC receiving paclitaxel, where patients experienced an increased hazard of death of 71% with one line of treatment before paclitaxel, a 30% increase with two prior treatments, and a 123% increase with three or more prior treatments compared to those with no prior treatments before paclitaxel [15]. Together these studies emphasize the importance of considering both duration and type of prior therapy when interpreting clinical trials results. These findings have implications for trial design and reporting for second- and later-line regimens, promoting the need for statistical analysis that takes heterogeneity of prior therapies into account. Furthermore, this has practical applications for physicians who need to convey prognostic information to patients and must be able to identify how individual patients in clinic are similar or different to the study population.

Another key finding of this study is the survival benefits observed for patients who switch CDK 4/6 inhibitors compared to those who receive subsequent endocrine, chemotherapy, and non-CDK4/6-targeted therapies. Our study finding of a 21% reduced hazard of death supports prior early findings regarding patients receiving multiple CDK4/6 inhibitors. Wander et al. found that patients who progressed to Abemaciclib after receiving Palbociclib had similar efficacy to the MONARCH-1 trial with a median progression-free survival of 5.3 months and a median overall survival on Abemaciclib of 17.2 months. [16, 17] This study was limited by a small sample size (*n* = 87) and only analyzed the relationship between switching from Palbociclib to Abemaciclib. In contrast, our study evaluated all combinations of CDK4/6 inhibitors in a substantially larger sample (*n* = 635). While these studies suggest benefit of continued CDK4/6 inhibition, further randomized studies are needed to definitively quantify the magnitude of benefit for second CDK4/6 inhibitor compared to other treatment strategies.

Given the challenge in capturing these higher dimensional data for different treatment sequences and their metadata, we employed our visualization algorithm to help create a topological representation of such heterogeneity [15, 18]. The features of the resultant treatment sequence graph can inform us about sequence frequency, median survival, overall average prior lines, and performance of any specific agent on disease behavior and outcome within the large sequence landscape of breast cancer. For example, by centering the zero time point on CDK4/6 agents, we can see the diverse outcome for our real-world patients. In part, this heterogeneity can be explained by impact of prior and subsequent treatments causing selective pressure with evolutionary expansion of resistant clones, such as CCNE1/2, RB1, and ERBB2 in case of CDK4/6 resistance [19]. Another feature of the graph is the inflection points of survival line, which would roughly describe variability of median survival across grouped sequences. For example, in Figure 1, we see an inflection point separating sequences that contain chemotherapy in the upper part from those that utilize further endocrine therapy below. These features attest to the power of our visualization solution and its adaptability and suitability for describing rather complex yet very important real-world outcomes of therapeutic interventions in breast cancer. Incorporating more higher dimensional data into our visualization tool, such as genomic drivers, intrinsic subtypes, and metastatic burden, can add further features and significant insight into the treatment landscape. The approach of leveraging real-world databases to evaluate regimens after clinical trials are completed can also be applied within cancer types where the paradigm includes sequential therapies. [20, 21]

There are important limitations to consider in this analysis. We were unable to account for comorbidities in our current analysis as current EHR International Classification of Diseases (ICD) codes are not sufficient in identifying comorbidities [22]. This may have overestimated the relationships seen on prior treatment duration and class and post-CDK 4/6 inhibitor survival. We were also unable to account for biological factors such as genetic tumor alterations which could have influenced OS if mutations developed shortened the post-CDK 4/6 inhibitor survival. We also did not focus on a specific CDK 4/6 inhibitor; therefore, we are unable to state whether these results are the same across each type. Similarly, when analyzing patients who switch CDK 4/6 inhibitors we did not differentiate between the order of CDK 4/6 inhibitors; therefore, further research is needed to determine if there is an optimal sequence for patients receiving multiple CDK 4/6 inhibitors. Considering the heterogeneity of MBC treatments, residual confounding may remain as we were unable to account for clinician-specific decision-making and all patient-specific characteristics that may play a role in treatment decisions.

## Conclusion

This study found that both duration and type of prior treatment before receipt of CDK 4/6 inhibitors impact survival after receipt of cancer therapy, highlighting the importance of treatment sequencing when interpreting survival outcomes. As additional treatment options are added to the MBC treatment milieu, it is crucial that treatment history in patient cohorts under study be analyzed and integrated in considered in their development to aid in integrating them with other treatment options.

## Supplementary Information

Below is the link to the electronic supplementary material.

Supplementary file1 (DOC 33 KB)

## Data Availability

Data that support the findings of this study have been originated by Flatiron Health, Inc. These de-identified data may be made available upon request and are subject to a license agreement with Flatiron Health; interested researchers should contact < DataAccess@flatiron.com > to determine licensing terms.

## Abbreviations

MBC: Metastatic breast cancer
HR+: Hormone receptor-positive
HER2−: Human epidermal growth factor 2-negative
CDK: Cyclin-dependent kinase
OS: Overall survival
IQR: Interquartile range
HR: Hazard ratio
CI: Confidence interval
PFS: Progression-free survival
EHR: Electronic health record
ICD: International classification of diseases
