# Supplementary material for: Effect of prior treatments on post-CDK 4/6 inhibitor survival in hormone receptor-positive breast cancer
Source: Breast Cancer Res Treat. 2022 Dec 21;197(3):673–81. doi: 10.1007/s10549-022-06823-w (PMC9883320; doi:10.1007/s10549-022-06823-w)
Supplement: Supplementary file 1 — Supplementary file1 (DOC 33 KB) [file 10549_2022_6823_MOESM1_ESM.doc]

**Title:** Effect of prior treatments on post-CDK 4/6 inhibitor survival

**Authors:** Jeffrey Franks, MSPH^1^; Nicole E. Caston, MPH^1^; Ahmed Elkhanany, MD^1, 2^; Travis Gerke, PhD ^3^; Andres Azuero, PhD, MBA ^2,4^; Gabrielle B. Rocque, MD, MSPH^1, 2^

**Author affiliations:**

^1^University of Alabama at Birmingham, Department of Medicine, Division of Hematology and Oncology; Birmingham, AL

^2^ O’Neal Comprehensive Cancer Center; Birmingham, AL

^3^The Prostate Cancer Clinical Trials Consortium; New York, NY

^4^ University of Alabama at Birmingham, School of Nursing; Birmingham, AL

**Corresponding author:**

Gabrielle Rocque, MD

University of Alabama at Birmingham

1824 6^th^ Avenue South, Birmingham, Al. 35924-3300 – WTI 240E

(205) 975-2914

grocque@uabmc.edu

| **Supplemental 1.** The association between OS and treatment type following the first CDK 4/6 inhibitor | | |
| --- | --- | --- |
| **Treatment following first CDK** | **Hazard ratio** | **95% Confidence interval** |
| **1-to-1 Matched analysis* (n= 984)** |  |  |
| Single CDK 4/6 inhibitor | - | - |
| Multiple CDK 4/6 inhibitor | 0.81 | 0.67 to 1.0 |
| **2- to-1 Matched analysis** (n=1278)** |  |  |
| Single CDK 4/6 inhibitor | - | - |
| Multiple CDK 4/6 inhibitor | 0.79 | 0.65 to 0.96 |
| Abbreviations: OS, Overall survival; CDK, cyclin-dependent kinase  All patients were diagnosed with metastatic breast cancer in 2011 – 2020  Hazard ratios and 95% confidence intervals obtained by Cox proportional hazard models  Propensity scores were created from age at diagnosis; race; site of metastasis; prior treatment duration (years); first CDK 4/6 inhibitor type; first CDK 4/6 inhibitor duration; additional matching by time alive and number of therapy lines  Patients were only included if they switched treatments after their first CDK 4/6 inhibitor  *n=492 patients who switched to a later-line endocrine or chemotherapy and n=492 patients with a second CDK 4/6 inhibitor  **n=852 patients who switched to a later-line endocrine or chemotherapy and n=426 patients with a second CDK 4/6 inhibitor | | |
